# Prefrontally mediated inhibition of memory systems in dissociative amnesia

**DOI:** 10.1017/S0033291724003040

**Published:** 2024-12

**Authors:** Laura C. Marsh, Dace Apšvalka, Hirokazu Kikuchi, Nobuhito Abe, Jun Kawaguchi, Michael D. Kopelman, Michael C. Anderson

**Affiliations:** 1 MRC Cognition and Brain Sciences Unit, University of Cambridge, Cambridge, UK; 2 Department of Neurology, Sendai Nakae Hospital, Sendai, Japan; 3 Institute for the Future of Human Society, Kyoto University, Kyoto, Sakyo-ku, Japan; 4 Department of Cognitive and Psychological Sciences, Graduate School of Informatics, Nagoya University, Nagoya, Japan; 5 Department of Academic Neuropsychiatry, Institute of Psychiatry, Psychology and Neuroscience, King's College London, London, UK

**Keywords:** dissociative amnesia, effective connectivity, memory inhibition, psychogenic amnesia, retrieval stopping, retrieval suppression

## Abstract

**Background:**

The mechanisms underlying generalized forms of dissociative (‘psychogenic’) amnesia are poorly understood. One theory suggests that memory retrieval is inhibited via prefrontal control. Findings from cognitive neuroscience offer a candidate mechanism for this proposed retrieval inhibition. By applying predictions based on these experimental findings, we examined the putative role of retrieval suppression in dissociative amnesia.

**Methods:**

We analyzed fMRI data from two previously reported cases of dissociative amnesia. Patients had been shown reminders from forgotten and remembered time periods (colleagues and school friends). We examined the neuroanatomical overlap between regions engaged in the unrecognized compared to the recognized condition, and the regions engaged during retrieval suppression in laboratory-based tasks. Effective connectivity analyses were performed to test the hypothesized modulatory relationship between the right anterior dorsolateral prefrontal cortex (raDLPFC) and the hippocampus. Both patients were scanned again following treatment, and analyses were repeated.

**Results:**

We observed substantial functional alignment between the inhibitory regions engaged during laboratory-based retrieval suppression tasks, and those engaged when patients failed to recognize their current colleagues. This included significant activation in the raDLPFC and right ventrolateral prefrontal cortex, and a corresponding deactivation across autobiographical memory regions (hippocampus, medial PFC). Dynamic causal modeling confirmed the hypothesized modulatory relationship between the raDLPFC and the hippocampus. This pattern was no longer evident following memory recovery in the first patient, but persisted in the second patient who remained amnesic.

**Conclusions:**

Findings are consistent with an inhibitory mechanism driving down activity across core memory regions to prevent the recognition of personally relevant stimuli.

## Introduction

Dissociative amnesia, also known as psychogenic or functional amnesia, refers to the loss of autobiographical memory, occurring in the absence of structural neurological damage, presumed to have a psychological cause. This can involve memory loss for traumatic experiences, such as in PTSD. It can also involve more extensive amnesia for several years or decades of a person's past (psychogenic focal retrograde amnesia), or, more extremely, fugue states, which involve a transient loss of all memory and personal identity, often with a period of wandering or travel away from home (Harrison et al., 2017; Staniloiu & Markowitsch, 2014). These latter, ‘generalized’ forms of dissociative amnesia are rare, and the underlying mechanisms are poorly understood.

Based on findings of reduced glucose metabolism across prefrontal and medial temporal regions implicated in autobiographical memory (Brand et al., 2009; Markowitsch et al., 1998; Markowitsch & Staniloiu, 2013), Markowitsch and colleagues (Markowitsch et al., 1999; Markowitsch & Staniloiu, 2013) have suggested that the memory loss results from a stress hormone mediated desynchronization and deactivation of fronto-temporal memory retrieval systems. Kopelman (2000, 2002, 2019) proposed that these memory systems may be deactivated by a prefrontally mediated inhibitory mechanism which prevents retrieval of memories from specific periods of the personal past. This theory is supported by findings of increased activity across right prefrontal control regions in response to reminders from forgotten periods of the personal past (e.g. Fujiwara et al., 2004; Kikuchi et al., 2010; Reinhold, Kuehnel, Brand, & Markowitsch, 2006).

A parallel body of experimental work has defined an inhibitory brain network engaged during the suppression of retrieval during laboratory-based tasks (Anderson & Green, 2001; Anderson & Hulbert, 2021; Anderson, Crespo-Garcia, & Subbulakshmi, 2024; Marsh & Anderson, 2024). This is typically studied using the ‘Think/No-Think task, in which participants learn cue-target associations, and are then instructed to suppress target recall for one subset of cues (‘No-Think’ trials), and to recall targets for the other subset (‘Think’ trials). Suppressing retrieval of ‘No-Think’ items is associated with the engagement of right-lateralized prefrontal regions, including the right anterior dorsolateral and ventrolateral prefrontal cortices (raDLPFC and rVLPFC), posterior middle frontal gyrus and insula (Anderson et al., 2004, 2016; Apšvalka, Ferreira, Schmitz, Rowe, & Anderson, 2022; Benoit, Hulbert, Huddleston, & Anderson, 2015; Guo, Schmitz, Mur, Ferreira, & Anderson, 2018). These prefrontal regions belong to a broader multi-modal inhibitory control network, with common regions of the raDLPFC, rVLPFC, precentral gyrus, supramarginal gyrus, and supplementary motor area engaged during both retrieval suppression and motor action cancellation (Apšvalka et al., 2022; Guo et al., 2018). During retrieval suppression, a corresponding decreased activation is typically observed in the hippocampi, and across wider cortical and sub-cortical regions in which the suppressed content is represented (e.g. amygdala for fearful memories, object-perception regions for object memories; Anderson et al., 2004, 2016; Benoit et al., 2015; Gagnepain, Henson, & Anderson, 2014; Gagnepain, Hulbert, & Anderson, 2017). Effective connectivity analyses have confirmed a modulatory relationship between the raDLPFC and the hippocampus during retrieval suppression (e.g. Apšvalka et al., 2022; Benoit & Anderson, 2012; Gagnepain et al., 2017). Indeed, negative coupling between these regions predicts later impaired recall of ‘No-Think’ items (Benoit & Anderson, 2012), a commonly observed behavioral aftereffect of retrieval suppression (Anderson & Green, 2001; Anderson & Hulbert, 2021).

These experimental findings offer a candidate neurobiological mechanism for the proposed inhibition of memory systems in dissociative amnesia and provide a set of testable predictions regarding the putative role of this mechanism in the memory loss (Harrison et al., 2017). The current study tested these predictions in a re-analysis of fMRI data from two patients with dissociative amnesia (Kikuchi et al., 2010). The original study reported activation across prefrontal control regions (bilateral DLPFC, VLPFC) when patients were reminded of people they could no longer recognize because of their amnesia. In the current study, we independently re-analyzed the data and examined (i) the neuroanatomical overlap with independently obtained networks of interest (retrieval suppression network), and (ii) activation changes in *a-priori* regions of interest (ROIs) defined based on retrieval suppression studies. We then conducted effective connectivity analyses to test the hypothesized modulatory relationship between right prefrontal and hippocampal regions.

We predicted the following:

- A neuroanatomical overlap between the specific regions engaged when patients were unable to recognize people from their past, and the network engaged during retrieval suppression, particularly the critical inhibitory raDLPFC region.
- A concurrent decrease in activation across critical autobiographical memory regions (hippocampus, medial prefrontal cortex) during failures to recognize people from the recent past.
- Evidence for a causal modulatory relationship between the raDLPFC and hippocampus, with negative coupling between the raDLPFC and the hippocampus indicating prefrontal downregulation of hippocampal activity in response to reminders from forgotten time-periods.
- Finally, that we would no longer see engagement of the retrieval stopping network following memory recovery, indicating a mechanistic role in the memory loss.

## Methods

Data were derived from a previously published study (Kikuchi et al., 2010), generously shared by the original authors. Data were independently pre-processed and analyzed.

### Participants

Full case descriptions and background neuropsychology is described in the original publication (Kikuchi et al., 2010). In brief, ‘patient 1’ was a 27-year-old man, who had been working in business for about 4 years after graduating from college. He woke up one morning not knowing who or where he was. He found his ID card in his wallet, from which he identified his name and profession, but he was not otherwise aware of these. This fugue episode lasted for approximately 2 days, following which he recovered most of his memories, but remained amnesic for the previous ~4 ½ years of his life. Patient 1 showed preserved neuropsychological functioning across all other domains, including attention, language, executive function, and anterograde memory. There were no other neurological symptoms, and all clinical investigations and scans showed normal results. Prior to the amnesia onset, patient 1 had been experiencing a very busy period at work and had been worried about an impending marriage. Patient 1 was seen approximately 1 month after the onset of his amnesia.

Patient 2 was a 52-year-old married man, who lost his memory following a minor car accident on the way to work, when he struck a guardrail. He was not injured, aside from a few bruises, and head CT was normal. He was given a brief leave of absence from work, during which he stayed at home, and had no problems in daily life. However, 1 month later, he was talking to a colleague on the phone, he realized that he knew nothing about the company, his job, or who he was talking to. It became apparent that he had a focal retrograde amnesia for the past 35 years of his life, recalling nothing since his graduation from high school. All physiological investigations and brain imaging were normal. Aside from this retrograde memory loss, more general neuropsychological functioning and anterograde memory was preserved. Personal antecedents to the amnesia included ongoing divorce negotiations and increasing debt. Patient 2 was seen a few months after onset.

Both patients were treated by conducting a memory interview while they were sedated with sodium thiopental. Following two interviews, patient 1 recovered almost all his memories, with a persisting amnesia for just the 6 months prior to the amnesia onset. By contrast, patient 2 did not recover any memories, and remained amnesic for the past 35 years of his past.

### Task stimuli and paradigm

The patients were each shown reminders of people from their own personal pasts. These included (i) reminders of high school friends who the patients still recognized (‘recalled’ condition), and (ii) reminders of current colleagues with whom the patients were well acquainted, but could not recognize because of their amnesia (‘forgotten’ condition). Stimuli included 12 face photographs and 12 names for each condition, obtained from the patients' family and colleagues, who verified that the patient had known the individuals depicted. A third condition involved people who had never been known to the patient, and a fourth scrambled face/name condition was included as a rest block. While in the scanner, patients were asked to make a yes/no recognition judgement for each stimulus. When they recognized a person, they were asked to continue looking at the stimulus, and to silently recall their relationship and events associated with them, to ensure that they were engaged in recollective processing for the full 4 s trial duration. Where the patient did not recognize the individual, they were asked to continue looking at the stimulus for the entire trial duration.

The task was completed across two fMRI runs (a face run and a name run). A blocked design was used to maximize the statistical power for single subject analysis. Blocks consisted of six faces or names, presented for 4 s each, with a 1 s inter-stimulus interval. Stimulus order was randomized within each block, and block order was counterbalanced within runs. Each block was repeated three times. Both patients completed this task during their amnesic state, prior to any treatment. The procedure was repeated following treatment, at which point patient 1 had recovered most of his memories, whereas patient 2 remained amnesic.^†^^1^

### fMRI data acquisition

Images were acquired using a 1.5-T General Electric Signa scanner. 1 × 1 × 1.5 mm resolution T1-weighted images were acquired at the start of the session. Functional volumes consisted of 26 axial slices (4 mm slice thickness, 1 mm interslice gap), obtained using T2*-weighted echo-planar imaging (EPI) sequence (repetition time = 2500 msec, echo time = 30 msec, flip angle = 90°, 64 × 64 acquisition matrix, field of view = 260 mm).

### fMRI data analysis

#### Pre-processing

Imaging data were analyzed using SPM12 (http://www.fil.ion.ucl.ac.uk/spm/) in MATLAB R2018a. Images were manually re-oriented to the anterior–posterior commissure line, and the origin was set to the anterior commissure. Pre-processing included spatial re-alignment, slice-time correction, co-registration, and segmentation. Post-treatment session images were co-registered to the mean pre-treatment session EPI to align across sessions.

#### Univariate whole-brain analysis

For the univariate analysis, EPI images were normalized to MNI space using DARTEL (Ashburner, 2007), and smoothed with a 10 × 10 × 10 mm Gaussian kernel. As per the original analysis, data were concatenated across the two stimulus types (names, faces) to increase statistical power. Given the use of a slow blocked design, a high-pass filter of 346 s (2x the maximum length between the same stimulus type) was applied to filter out low-frequency noise. The pre-processed time-series data were entered into a first-level general linear model. The two conditions of interest (recognized high school, unrecognized colleague) were modeled as boxcar functions convolved with a canonical hemodynamic response function. The control conditions (scrambled and novel) were left in the baseline. Realignment parameters were included as regressors of no interest to account for movement artefacts, along with a constant for each task type (names, faces). The height threshold was set at *p* < 0.001 uncorrected, with a spatial threshold of five contiguous voxels.

We examined the neuroanatomical overlap between the regions engaged when patients were reminded of forgotten colleagues, and the network of regions typically engaged during retrieval suppression. The canonical retrieval suppression network was derived from an independent map of brain areas involved in both retrieval stopping and action stopping. This was obtained from a meta-analysis of Stop signal (Stop > Go) and Think/No-Think (No-think > Think) tasks based on 56 studies (Apšvalka et al., 2022). The conjunction between retrieval stopping and action stopping was used in preference to the simple No-Think > Think network to isolate the regions specifically involved in inhibitory control across the two tasks. This map was overlaid with the contrasts of the unrecognized colleague > recognized high school conditions to allow visual inspection of the overlap.

#### Regions of interest

We defined a set of ROIs for univariate and dynamic causal modeling (DCM) analyses. The raDLPFC region from the aforementioned meta-analytic conjunction of retrieval stopping and action stopping was isolated from the broader network, as engagement of this region is particularly emphasized in retrieval suppression research (e.g. Anderson et al., 2004, 2016; Apšvalka et al., 2022; Depue, Curran, & Banich, 2007; Gagnepain et al., 2017), and has been the focus of previous effective connectivity analyses of retrieval suppression (Benoit et al., 2015, 2016; Benoit & Anderson, 2012; Gagnepain et al., 2017; Schmitz, Correia, Ferreira, Prescot, & Anderson, 2017).

Hippocampal ROIs were obtained by manually segmenting the T1-weighted images using ITK-SNAP (www.itksnap.org; Yushkevich et al., 2006), following established guidelines (Harmonised Hippocampal Protocol; Boccardi et al., 2015). Evidence suggests distinct roles of hippocampal sub-regions during memory retrieval (e.g. Sheldon & Levine, 2016). Thus, we generated a heatmap of the hippocampal voxels which were most frequently deactivated during retrieval suppression, based on an unpublished mega-analysis of data from 10 Think/No-Think studies (330 participants; No-Think < Think and No-Think < implicit baseline [i.e. unmodeled data], *p* < 0.05 uncorrected). For each patient, the voxels that were significantly deactivated in the unrecognized compared to recognized conditions were superimposed on the mega-analytic heatmap, allowing visual inspection of the overlap.

Finally, we defined an autobiographical memory retrieval network of interest, derived from a previous meta-analysis of 14 studies conducted by McDermott, Szpunar, and Christ (2009), made publicly available by Chow, Westphal, and Rissman (2018) (https://neurovault.org/collections/3412/). This included the left medial prefrontal cortex, anterior cingulate, supplementary motor area, posterior cingulate/retrosplenial cortex, angular gyrus, cuneus and precuneus, and bilateral medial temporal lobes (hippocampus and parahippocampal gyri). Because of the particularly important role of the mPFC in the retrieval of temporally remote autobiographical memories (McCormick, Barry, Jafarian, Barnes, & Maguire, 2020), we also extracted the mPFC region from the broader autobiographical memory network as a separate, more specific ROI.

ROI and DCM analyses were conducted on unsmoothed images in each patient's native space to maximize the anatomical specificity of hand-traced hippocampal ROIs. ROI masks which were created in MNI space (rDLPFC, autobiographical memory network, mPFC) were projected back into participants' native spaces using inversed normalization parameters. ROI analyses were conducted using MarsBar (https://marsbar-toolbox.github.io/index.html). Percent signal change was extracted for task conditions (high school, colleague), relative to baseline conditions. One-tailed paired *t* tests were used to compare activation across task conditions within each patient (*p* < 0.05 uncorrected). These statistical comparisons are equivalent to the statistical comparisons made at the whole-brain level, both of which have limited power due to the use of a single subject design.

#### Effective connectivity

DCM is a computational framework that allows one to make inferences regarding the causal modulatory influences (effective connectivity) between brain regions (Friston, Harrison, & Penny, 2003). Hypothesized interactions between specified brain areas are used to generate possible models, defined in terms of driving input, intrinsic connectivity between regions, and condition-based modulation. These models are combined with a hemodynamic forward model and fitted to the data, allowing comparison of model fit. We applied DCM to confirm whether there was top-down modulation of the right hippocampus by the raDLPFC when patients viewed photographs of unrecognized colleagues.

For the raDLPFC ROI, we extracted the time-series for voxels that were significantly more active in the unrecognized colleague relative to the recognized high school condition (*p* < 0.05, uncorrected). For the right hippocampus, we extracted voxels in which activation was significantly reduced in the unrecognized condition, relative to both the recognized condition and the implicit baseline (*p* < 0.05, uncorrected). The right hippocampus was selected for consistency with previous DCM analyses of the Think/No-Think task and given the model complexity cost in Bayesian model selection (BMS; Benoit et al., 2015; Benoit & Anderson, 2012; Gagnepain et al., 2014).

We constructed a set of 12 models, comprised of two nodes (raDLPFC, right hippocampus), with full intrinsic connectivity, allowing for all possible combinations of modulatory connections and driving inputs, including a null model. Models were grouped into families with no modulatory relationship, top-down modulation, bottom-up modulation, or bi-directional modulation, with driving input from the right hippocampus, raDLPFC, or from both regions. These models were inverted to fit the experimental data, and the relative model evidences were compared using BMS in a fixed-effects analysis. The degree of evidence in support of each model indicates the relative probability that the observed data were generated by that model, whilst accounting for model complexity. This was estimated for all 12 individual models, as well as for each model family. After establishing the model of connectivity which best fitted the data, we extracted DCM coupling parameters across all models, and performed Bayesian model averaging to establish the direction (negative or positive) and strength of the modulatory relationship during the colleague condition. This involved computing the average of each model parameter, weighted according to the posterior probability of each model. The resulting coupling parameters quantify the modulatory influence between the raDLPFC and the right hippocampus.

## Results

### Reminders from forgotten time-periods triggered activity across the retrieval stopping network

**Figure 1.**
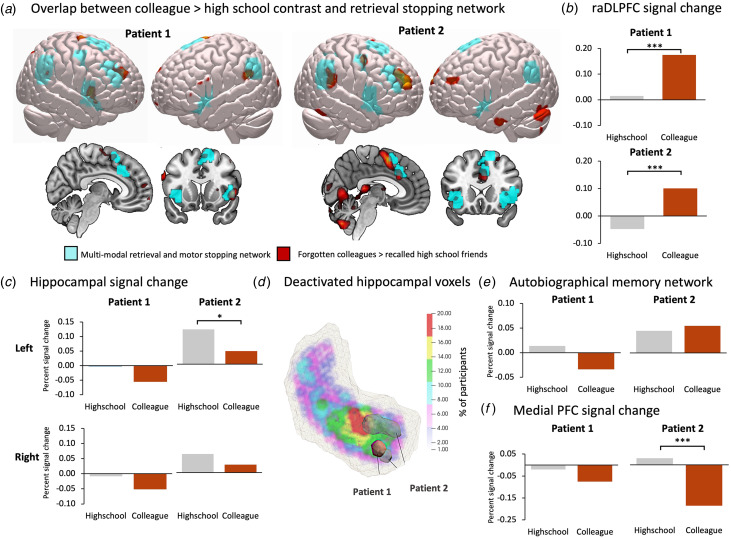
(*a*) Regions engaged during the failure to recognize current colleagues, relative to successful recognition of high school friends (red; *p* < 0.001), overlaid with the meta-analytic conjunction of retrieval and action stopping (blue), for each patient. (*b*) Percent signal change in the right anterior DLPFC ROI derived from the meta-analytic conjunction, showing significantly greater signal in the unrecognized colleague relative to recognized high school condition. (*c*) Percent signal change in the left and right hippocampus, showing reduced signal during the unrecognized colleague relative to recognized high school condition. (*d*) Heat map of right hippocampal voxels which most frequently showed reduced activation in the No-Think condition relative to implicit baseline, derived from a mega-analysis of 330 participants. Overlaid are the hippocampal voxels within each patient which showed significantly reduced activation in the forgotten colleague relative to remembered high school condition. (*e*) Percent signal change across the meta-analytic autobiographical memory network derived from McDermott et al. (2009), and (*f*) percent signal change within the mPFC region of the autobiographical memory network, showing reduced signal in the unrecognized colleague relative to recognized high school condition.

**Figure 2.**
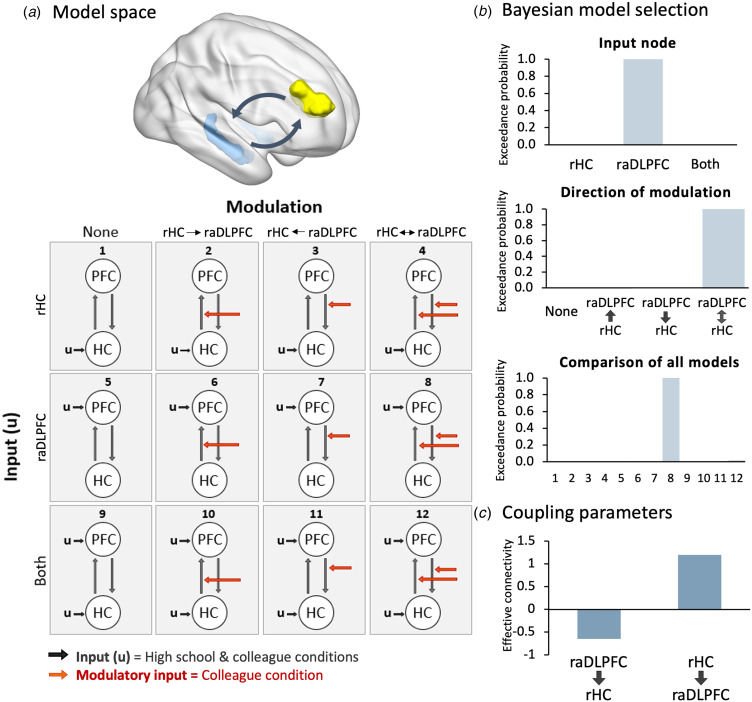
Dynamic causal modeling of the relationship between the raDLPFC (‘PFC’) and the right hippocampus (‘HC’) during the failure to recognize current colleagues. (*a*) The model space, derived from Benoit and Anderson (2012), included model families involving (i) no modulation, (ii) bottom-up modulation (iii) top-down modulation, and (iv) bi-directional modulation; with driving input to the (i) right hippocampus (HC), (ii) raDLPFC (PFC), or (iii) both regions. (*b*) The relative evidence (exceedance probability) for each input family; each modulation family and across all individual models, derived from Bayesian model selection. Evidence overwhelmingly favored the model involving bi-directional modulation (model 8). (*c*) Coupling parameters of the connection between the raDLPFC and right hippocampus during the colleague condition, derived from Bayesian model averaging of parameter estimates across the winning modulation family. Data are represented as the mean across the two patients, because the same pattern was observed in each patient separately (see online Supplementary material).

We observed activation in the raDLPFC and rVLPFC when patients viewed reminders of current colleagues, relative to high school friends, replicating the results originally reported by Kikuchi et al. (2010). There was substantial overlap between these activation maps and the inhibitory network engaged during retrieval suppression (meta-analytic conjunction of No-Think > Think and Stop > Go), including in the raDLPFC, bilateral VLPFC and insulae, mid-cingulate, right supplementary motor area, right precentral gyrus, and right supramarginal gyrus (Fig. 1a). Thus, both patients appeared to engage this core retrieval stopping network in response to reminders of forgotten colleagues. Consistent with this impression, both patients showed significantly greater BOLD signal in the raDLPFC ROI during the unrecognized colleague relative to the recognized high school conditions (patient 1: *t* = 3.49, *p* < 0.001; patient 2: *t* = 5.62, *p* < 0.001; Fig. 2b).

### Reduced activity across autobiographical memory regions

In addition to the engagement of prefrontal control regions, mean BOLD signal was reduced in the patients' hippocampal ROIs during the unrecognized colleague relative to recognized high school conditions (Fig. 1c). This decrease was statistically significant only in the left hippocampus of patient 2 (*t* = 1.79, *p* = 0.037; all other *p*s > 0.05), although the same pattern arose across hippocampal ROIs in both patients. Moreover, within the hippocampus, the specific voxels that showed significantly reduced activation in the unrecognized relative to the recognized condition substantially overlapped with those voxels most frequently downregulated in studies of voluntary retrieval stopping. Figure 1d shows the heatmap of the voxels in the right hippocampus which were most frequently deactivated during the No-Think relative to Think and implicit baseline (*p* < 0.05), derived from the Think/No-Think task mega-analysis (*n* = 330). The voxels showing significantly reduced activation during the colleague condition in each patient are superimposed on this heatmap, showing the substantial overlap within the anterior portion of the right hippocampus.

Regarding activity across the broader autobiographical memory regions, patient 1 showed a near-significant deactivation across the entire meta-analytic autobiographical memory network in the colleague relative to the high school condition (*t* = 1.53, *p* = 0.063), whereas no significant difference was observed in patient 2 (*t* = −0.43, *p* = 0.665; Fig. 1e). However, both patients showed reduced activity in the key mPFC region during the colleague relative to high school condition, with the decrease reaching significance in patient 2 (*t* = 3.37, *p* < 0.001), but not patient 1 (*t* = 1.03, *p* = 0.151; Fig. 1f).

### Hippocampal activity was modulated by the raDLPFC

Critical to our hypothesis was whether top-down inhibitory control regions (raDLPFC) modulated activity in memory systems (hippocampus) during the forgotten colleague condition. We used DCM to examine the effective connectivity between these regions during the forgotten colleague condition. Results are reported for the two patients combined, because the findings were consistent across both patients (see online Supplementary material).

BMS unambiguously favored models with bi-directional connectivity between the raDLPFC and hippocampus (exceedance probability = 0.9987). Comparing families with driving input to the hippocampus, raDLPFC or both nodes, BMS indicated clear evidence for model families involving driving input to the raDLPFC (exceedance probability = 0.9988). Consistent with these results, when all 12 individual models were compared there was unambiguous evidence favoring a single model (model 8) involving bi-directional modulation, with driving input to the raDLPFC (exceedance probability = 0.9987, Fig. 2b). Bayesian model averaging of the coupling parameters across models in the winning modulatory family confirmed a negative coupling between the raDLPFC and the right hippocampus, indicating prefrontally mediated downregulation of the hippocampus (Fig. 2c). Consistent with the modulatory relationship observed during laboratory-based retrieval suppression tasks, we also observed positive bottom-up coupling between the right hippocampus and raDLPFC.

### Memory recovery was associated with disengagement of inhibitory control

**Figure 3.**
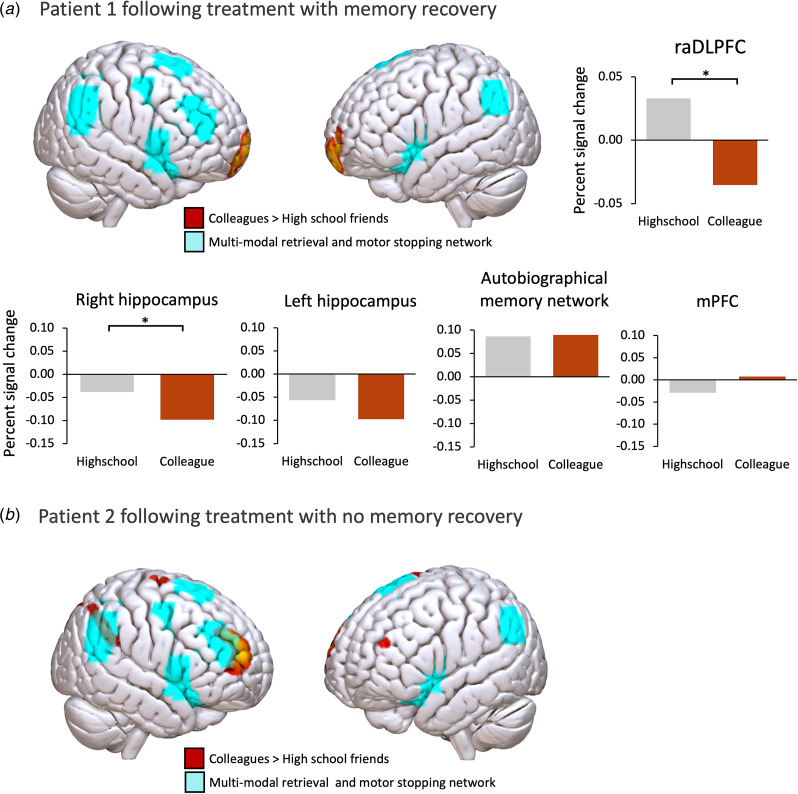
Pattern of neural activation on repeating the personal memory fMRI task following treatment. (*a*) Following recovery of his memories, patient 1 no longer showed engagement of regions overlapping with the multi-modal inhibition network when he was reminded of his current colleagues. Region of interest analyses revealed reduced raDLFPC activation in the colleague relative to high school condition. Hippocampal activation was still slightly reduced in the current colleague relative to high school conditions, but the broader autobiographical memory network and mPFC were similarly activated across conditions. (*b*) Patient 2 did not recover his memories following treatment, and the pattern of activation during the task remained much the same.

Both patients completed the fMRI task a second time following treatment for their memory loss. Patient 1 was able to recognize all his colleagues, and no longer showed engagement of the multi-modal inhibition network (Fig. 3a). Activity in the raDLFPC ROI showed a reversal of the pattern observed during the amnesic state: raDLPFC activation was significantly *reduced* in the colleague relative to the high school condition (*t* = 1.68, *p* = 0.047). Activity across the hippocampi remained slightly lower in the colleague relative to high school condition (left *t* = 0.99, *p* = 0.162, right *t* = 1.86, *p* = 0.032), whereas a non-significant increase in activity was seen across the autobiographical memory network and medial prefrontal cortex (AM network, *t* = −0.93, *p* = 0.822, mPFC *t* = −0.63, *p* = 0.735, Fig. 3a).

Patient 2 did not recover any of his memories following treatment, and the pattern of activation was much the same as before treatment (Fig. 3b), with significantly greater activation in the raDLPFC (*t* = 3.67, *p* < 0.001), and a reduction in hippocampal activation in the colleague condition that was not significant (*p*s > 0.05).

## Discussion

The current study applied predictions derived from experimental studies to test the hypothesized role of a prefrontally mediated retrieval stopping mechanism underlying dissociative amnesia. We observed high functional alignment between the inhibitory regions engaged during retrieval suppression, and those engaged when patients failed to recognize their current colleagues. This was associated with reduced activation across bilateral hippocampi (both patients), medial prefrontal cortex (both patients), and broader autobiographical memory network (patient 1). Further functional alignment was observed between the anterior voxels of the right hippocampus that were deactivated in the unrecognized colleague relative to the recognized high school condition, and those voxels most frequently deactivated during deliberate retrieval suppression. Critically, effective connectivity analyses indicated that the raDLPFC exerted a negative modulatory influence over right hippocampal activity during the forgotten colleague condition. These findings are consistent with an inhibitory control mechanism driving down activity across core memory regions to prevent the recognition of personally relevant stimuli. Notably, memory recovery was associated with disengagement of the retrieval suppression network, providing compelling evidence for its role in producing the amnesic state.

The observed downregulation of voxels in the anterior portion of the right hippocampus, and mPFC, suggests an inhibitory process targeting the early stages of memory retrieval. The anterior portion of the hippocampus has been implicated in the initiation and coordination of neural activity across the core autobiographical memory network, whereas posterior hippocampal regions are more involved in elaboration of fine-grained perceptual details (McCormick, St-Laurent, Ty, Valiante, & McAndrews, 2015; Sheldon & Levine, 2016; Zeidman & Maguire, 2016). The mPFC is similarly involved in initiating and coordinating episodic construction, particularly for temporally remote memories (Bonnici & Maguire, 2018; McCormick, Ciaramelli, De Luca, & Maguire, 2018), and, notably, is similarly deactivated during the deliberate suppression of episodic material (Benoit, Davies, & Anderson, 2016; Lu, Yang, & Qiu, 2023).

Consistent with laboratory-based retrieval suppression, the modulatory relationship observed between the raDLPFC and hippocampus during the failure to recognize current colleagues was bi-directional. The positive coupling between the hippocampus and raDLFPC is believed to reflect a process of prefrontal upregulation triggered by the activation of an unwelcome memory trace. In the context of dissociative amnesia, it is presumed that these inhibitory control mechanisms are engaged sub- or semi-consciously. Although the experimental literature has typically examined retrieval suppression under direct instruction, recent evidence has suggested that these processes may be engaged without conscious intent or awareness. In a subliminal cueing paradigm, Salvador et al. (2018) found that the later recall of words presented after sub-consciously perceived ‘No-Think’ cues was impaired, relative to baseline words (see also, Van Gaal, Lamme, Fahrenfort, & Ridderinkhof, 2011; Van Gaal, Ridderinkhof, Scholte, & Lamme, 2010; van Gaal, Ridderinkhof, van den Wildenberg, & Lamme, 2009 for evidence of subliminally cued motor inhibition). These findings suggest that retrieval suppression mechanisms may be engaged without conscious intent, consistent with their hypothesized role in dissociative amnesia (Harrison et al., 2017).

### Strengths and limitations

Diagnosis of dissociative amnesia can be challenging, and, as with many functional neurological symptoms, it can be challenging to conclusively rule out the possibility of feigning. However, in focal retrograde forms of dissociative amnesia, it is not unusual for symptoms to persist (Harrison et al., 2017; Hennig-Fast et al., 2008; Serra, Fadda, Buccione, Caltagirone, & Carlesimo, 2007). Both the current patients both showed a selective impairment in retrograde autobiographical memory, with preserved neurocognitive functioning across standardized tests of attention, language, executive functioning, and anterograde memory. Further, when patient 1 was asked to feign amnesia for previously forgotten faces following recovery, Kikuchi et al. (2010) reported a pattern of activation distinct from that observed during the amnesic state, involving left-lateralized DLPFC and bilateral VLPFC activation with no activation observed in the critical right anterior DLPFC region, and no hippocampal deactivation. These findings are consistent with prior reports of left-lateralized prefrontal activation during feigned memory impairment (e.g. Chen et al., 2015; Liang et al., 2012) and suggest that feigned memory impairment may have distinct neural correlates.

Another limitation of the current study is the sample of just two cases. Replications in further cases or larger group samples, with appropriate control groups, are now needed. Nonetheless, the results observed within these individuals strongly align with our *a-priori* predictions, and thus remain persuasive.

Only the right hippocampus was included in the models evaluated in the DCM analysis, for consistency with prior effective connectivity analysis of the Think/No Think task, and because including the left hippocampus and the mPFC as additional nodes would add unnecessary model complexity costs during BMS. However, the current findings provide evidence to support inclusion of these nodes in future connectivity analyses.

Further, while prior research has identified a range of psychosocial precipitants of dissociative amnesia (e.g. Harrison et al., 2017), and the current findings point to the specific neurobiological pathways underlying the prevention of retrieval in dissociative amnesia, further research is needed to understand exactly how these mechanisms become aberrantly engaged.

## Conclusions

The current findings provide compelling evidence for an inhibitory control mechanism operating to prevent autobiographical memory retrieval in dissociative amnesia, with right prefrontal regions downregulating hippocampal activity. Disengagement of this inhibitory control network was associated with memory recovery, indicating a mechanistic role in producing the amnesic state, and pointing to potential targets for future treatment.

## Supporting information

Marsh et al. supplementary material Marsh et al. supplementary material

## Footnotes

† The notes appear after the main text.

## Note

1 Following his memory recovery, patient 1 completed a third version of the task in which he was asked to feign amnesia for the previously forgotten colleagues. This is reported in the original publication, and is not re-reported here because no further analyses were conducted.

## Supplementary material

The supplementary material for this article can be found at https://doi.org/10.1017/S0033291724003040.

## References

[ref4] Anderson, M. C., Bunce, J. G., & Barbas, H. (2016). Prefrontal–hippocampal pathways underlying inhibitory control over memory. Neurobiology of Learning and Memory, 134(Part A), 145–161. 10.1016/j.nlm.2015.11.008 26642918 PMC5106245

[ref1] Anderson, M. C., & Green, C. (2001). Suppressing unwanted memories by executive control. Nature, 410(6826), 366–369. 10.1038/35066572 11268212

[ref2] Anderson, M. C., & Hulbert, J. C. (2021). Active forgetting: Adaptation of memory by prefrontal control. Annual Review of Psychology, 72(1), 1–36. 10.1146/annurev-psych-072720-094140 32928060

[ref3] Anderson, M. C., Ochsner, K. N., Kuhl, B., Cooper, J., Robertson, E., Gabrieli, S. W., … Gabrieli, J. D. E. (2004). Neural systems underlying the suppression of unwanted memories. Science, 303(5655), 232–235. 10.1126/science.1089504 14716015

[ref5] Anderson, M., Crespo-Garcia, M., & Subbulakshmi, S. (2024). Brain mechanisms underlying the inhibitory control of thought. 10.31234/osf.io/4wcnb 40379896

[ref6] Apšvalka, D., Ferreira, C. S., Schmitz, T. W., Rowe, J. B., & Anderson, M. C. (2022). Dynamic targeting enables domain-general inhibitory control over action and thought by the prefrontal cortex. Nature Communications, 13(1), 1–21. 10.1038/s41467-021-27926-w PMC8755760 35022447

[ref7] Ashburner, J. (2007). A fast diffeomorphic image registration algorithm. NeuroImage, 38(1), 95–113. 10.1016/j.neuroimage.2007.07.007 17761438

[ref8] Benoit, R. G., & Anderson, M. C. (2012). Opposing mechanisms support the voluntary forgetting of unwanted memories. Neuron, 76(2), 450–460. 10.1016/J.NEURON.2012.07.025 23083745 PMC3480638

[ref10] Benoit, R. G., Davies, D. J., & Anderson, M. C. (2016). Reducing future fears by suppressing the brain mechanisms underlying episodic simulation. Proceedings of the National Academy of Sciences of the USA, 113(52), E8492. 10.1073/PNAS.1606604114 27965391 PMC5206570

[ref9] Benoit, R. G., Hulbert, J. C., Huddleston, E., & Anderson, M. C. (2015). Adaptive top–down suppression of hippocampal activity and the purging of intrusive memories from consciousness. Journal of Cognitive Neuroscience, 27(1), 96–111. 10.1162/jocn_a_00696 25100219

[ref16] Boccardi, M., Bocchetta, M., Apostolova, L. G., Barnes, J., Bartzokis, G., Corbetta, G., … EADC-ADNI Working Group on the Harmonized Protocol for Manual Hippocampal Segmentation. (2015). Delphi definition of the EADC-ADNI Harmonized Protocol for hippocampal segmentation on magnetic resonance. Alzheimer's & Dementia, 11(2), 126–138. 10.1016/j.jalz.2014.02.009 PMC4419736 25130658

[ref11] Bonnici, H. M., & Maguire, E. A. (2018). Two years later – revisiting autobiographical memory representations in vmPFC and hippocampus. Neuropsychologia, 110, 159–169. 10.1016/J.NEUROPSYCHOLOGIA.2017.05.014 28502632 PMC5825381

[ref12] Brand, M., Eggers, C., Reinhold, N., Fujiwara, E., Kessler, J., Heiss, W. D., & Markowitsch, H. J. (2009). Functional brain imaging in 14 patients with dissociative amnesia reveals right inferolateral prefrontal hypometabolism. Psychiatry Research – Neuroimaging, 174(1), 32–39. 10.1016/j.pscychresns.2009.03.008 19783409

[ref13] Chen, Z. X., Xue, L., Liang, C. Y., Wang, L. L., Mei, W., Zhang, Q., & Zhao, H. (2015). Specific marker of feigned memory impairment: The activation of left superior frontal gyrus. Journal of Forensic and Legal Medicine, 36, 164–171. doi: 10.1016/j.jflm.2015.09.008, Epub 2015 Sep 12. PMID: 26479324. 26479324

[ref14] Chow, T. E., Westphal, A. J., & Rissman, J. (2018). Multi-voxel pattern classification differentiates personally experienced event memories from secondhand event knowledge. NeuroImage, 176, 110–123. 10.1016/J.NEUROIMAGE.2018.04.024 29654876

[ref15] Depue, B. E., Curran, T., & Banich, M. T. (2007). Prefrontal regions orchestrate suppression of emotional memories via a two-phase process. Science, 317, 215–219. 10.1126/science.1139560 17626877

[ref17] Friston, K. J., Harrison, L., & Penny, W. (2003). Dynamic causal modelling. NeuroImage, 19(4), 1273–1302. 10.1016/S1053-8119(03)00202-7 12948688

[ref18] Fujiwara, E., Piefke, M. L. S., Fink, G. R., Kessler, J., Kracht, L., … Markowitsch, H. J. (2004). Brain correlates of functional retrograde amnesia in three patients. Brain and Cognition, 54(2), 135–176. 10.1016/S0278-2626(03)00268-9 15022660

[ref19] Gagnepain, P., Henson, R. N., & Anderson, M. C. (2014). Suppressing unwanted memories reduces their unconscious influence via targeted cortical inhibition. Proceedings of the National Academy of Sciences, 111(13), E1310–E1319. 10.1073/PNAS.1311468111 PMC3977236 24639546

[ref20] Gagnepain, P., Hulbert, J., & Anderson, M. C. (2017). Parallel regulation of memory and emotion supports the suppression of intrusive memories. Journal of Neuroscience, 37(27), 6423–6441. 10.1523/JNEUROSCI.2732-16.2017 28559378 PMC5511877

[ref21] Guo, Y., Schmitz, T. W., Mur, M., Ferreira, C. S., & Anderson, M. C. (2018). A supramodal role of the basal ganglia in memory and motor inhibition: Meta-analytic evidence. Neuropsychologia, 108, 117–134. 10.1016/J.NEUROPSYCHOLOGIA.2017.11.033 29199109 PMC5759998

[ref22] Harrison, N. A., Johnston, K., Corno, F., Casey, S. J., Friedner, K., Humphreys, K., … Kopelman, M. D. (2017). Psychogenic amnesia: Syndromes, outcome, and patterns of retrograde amnesia. Brain, 140(9), 2498–2510. 10.1093/brain/awx186 29050391

[ref23] Hennig-Fast, K., Meister, F., Frodl, T., Beraldi, A., Padberg, F., Engel, R. R., … Meindl, T. (2008). A case of persistent retrograde amnesia following a dissociative fugue: Neuropsychological and neurofunctional underpinnings of loss of autobiographical memory and self-awareness. Neuropsychologia, 46(12), 2993–3005. doi: 10.1016/j.neuropsychologia.2008.06.014, Epub 2008 Jun 21. PMID: 18619985. 18619985

[ref24] Kikuchi, H., Fujii, T., Abe, N., Suzuki, M., Takagi, M., Mugikura, S., … Mori, E. (2010). Memory repression: Brain mechanisms underlying dissociative amnesia. Journal of Cognitive Neuroscience, 22(3), 602–613. 10.1162/jocn.2009.21212 19301997

[ref25] Kopelman, M. D. (2000). Focal retrograde amnesia and the attribution of causality: An exceptionally critical review. Cognitive Neuropsychology, 17(7), 585–621. 10.1080/026432900750002172 20945196

[ref26] Kopelman, M. D. (2002). Disorders of memory. Brain, 125(10), 2152–2190. 10.1093/brain/awf229 12244076

[ref27] Kopelman, M. D. (2019). Anomalies of autobiographical memory. Journal of the International Neuropsychological Society, 25(10), 1061–1075. 10.1017/S135561771900081X 31474234

[ref28] Liang, C. Y., Xu, Z. Y., Mei, W., Wang, L. L., Xue, L., Lu, D. J., & Zhao, H. (2012). Neural correlates of feigned memory impairment are distinguishable from answering randomly and answering incorrectly: An fMRI and behavioral study. Brain and Cognition, 79(1), 70–77. doi: 10.1016/j.bandc.2012.01.009, Epub 2012 Feb 22. PMID: 22361169. 22361169

[ref29] Lu, F. Y., Yang, W. J., & Qiu, J. (2023). Neural bases of motivated forgetting of autobiographical memories. Cognitive Neuroscience, 14(1), 15–24. 10.1080/17588928.2022.2136150 36409182

[ref32] Markowitsch, H. J., Kessler, J., Russ, M. O., Frölich, L., Schneider, B., & Maurer, K. (1999). Mnestic block syndrome. Cortex, 35(2), 219–230. 10.1016/S0010-9452(08)70795-0 10369094

[ref31] Markowitsch, H. J., Kessler, J., Van der Ven, C., Weber-Luxenburger, G., Albers, M., & Heiss, W. D. (1998). Psychic trauma causing grossly reduced brain metabolism and cognitive deterioration. Neuropsychologia, 36(1), 77–82. 9533390 10.1016/s0028-3932(97)00093-6

[ref30] Markowitsch, H. J., & Staniloiu, A. (2013). The impairment of recollection in functional amnesic states. Cortex, 49(6), 1494–1510. 10.1016/j.cortex.2012.05.020 22824728

[ref33] Marsh, L. C., & Anderson, M. C. (2024) Inhibition as a cause of forgetting. In M. J. Kahana & A. D. Wagner (Eds.), The Oxford handbook of human memory, two volume pack: Foundations and applications (pp. 1209–1256). New York: Oxford University Press. 10.1093/oxfordhb/9780190917982.013.41

[ref36] McCormick, C., Barry, D. N., Jafarian, A., Barnes, G. R., & Maguire, E. A. (2020). vmPFC drives hippocampal processing during autobiographical memory recall regardless of remoteness. Cerebral Cortex, 30(11), 5972–5987. 10.1093/cercor/bhaa172 32572443 PMC7899055

[ref35] McCormick, C., Ciaramelli, E., De Luca, F., & Maguire, E. A. (2018). Comparing and contrasting the cognitive effects of hippocampal and ventromedial prefrontal cortex damage: A review of human lesion studies. Neuroscience, 374, 295–318. 10.1016/J.NEUROSCIENCE.2017.07.066 28827088 PMC6053620

[ref34] McCormick, C., St-Laurent, M., Ty, A., Valiante, T. A., & McAndrews, M. P. (2015). Functional and effective hippocampal–neocortical connectivity during construction and elaboration of autobiographical memory retrieval. Cerebral Cortex, 25(5), 1297–1305. 10.1093/CERCOR/BHT324 24275829 PMC4397572

[ref37] McDermott, K. B., Szpunar, K. K., & Christ, S. E. (2009). Laboratory-based and autobiographical retrieval tasks differ substantially in their neural substrates. Neuropsychologia, 47(11), 2290–2298. 10.1016/J.NEUROPSYCHOLOGIA.2008.12.025 19159634

[ref38] Reinhold, N., Kuehnel, S., Brand, M., & Markowitsch, H. J. (2006). Neuroimaging in memory and memory disturbances. Current Medical Imaging Reviews, 2, 35–57.

[ref39] Salvador, A., Berkovitch, L., Vinckier, F., Cohen, L., Naccache, L., Dehaene, S., & Gaillard, R. (2018). Unconscious memory suppression. Cognition, 180, 191–199. 10.1016/j.cognition.2018.06.023 30075345

[ref40] Schmitz, T. W., Correia, M. M., Ferreira, C. S., Prescot, A. P., & Anderson, M. C. (2017). Hippocampal GABA enables inhibitory control over unwanted thoughts. Nature Communications, 8(1), 1–11. 10.1038/s41467-017-00956-z PMC5670182 29101315

[ref41] Serra, L., Fadda, L., Buccione, I., Caltagirone, C., & Carlesimo, G. A. (2007). Psychogenic and organic amnesia. A multidimensional assessment of clinical, neuroradiology, neuropsychological and psychopathological features. Behavioural Neurology, 18(1), 53–64. 10.1155/2007/193140 17297220 PMC5469968

[ref42] Sheldon, S., & Levine, B. (2016). The role of the hippocampus in memory and mental construction. Annals of the New York Academy of Sciences, 1369(1), 76–92. 10.1111/nyas.13006 26849289

[ref43] Staniloiu, A., & Markowitsch, H. J. (2014). Dissociative amnesia. The Lancet Psychiatry, 1(3), 226–241. 10.1016/S2215-0366(14)70279-2 26360734

[ref46] Van Gaal, S., Lamme, V. A. F., Fahrenfort, J. J., & Ridderinkhof, K. R. (2011). Dissociable brain mechanisms underlying the conscious and unconscious control of behavior. Journal of Cognitive Neuroscience, 23(1), 91–105. 10.1162/JOCN.2010.21431 20175675

[ref45] Van Gaal, S., Ridderinkhof, K. R., Scholte, H. S., & Lamme, V. A. F. (2010). Unconscious activation of the prefrontal no-go network. Journal of Neuroscience, 30(11), 4143–4150. 10.1523/JNEUROSCI.2992-09.2010 20237284 PMC6632275

[ref44] van Gaal, S., Ridderinkhof, K. R., van den Wildenberg, W. P. M., & Lamme, V. A. F. (2009). Dissociating consciousness from inhibitory control: Evidence for unconsciously triggered response inhibition in the stop-signal task. Journal of Experimental Psychology. Human Perception and Performance, 35(4), 1129–1139. 10.1037/A0013551 19653754

[ref47] Yushkevich, P. A., Piven, J., Hazlett, H. C., Smith, R. G., Ho, S., Gee, J. C., & Gerig, G. (2006). User-guided 3D active contour segmentation of anatomical structures: Significantly improved efficiency and reliability. NeuroImage, 31(3), 1116–1128. 10.1016/j.neuroimage.2006.01.015 16545965

[ref48] Zeidman, P., & Maguire, E. A. (2016). Anterior hippocampus: The anatomy of perception, imagination and episodic memory. Nature Reviews Neuroscience, 17(3), 173–182. 10.1038/nrn.2015.24 26865022 PMC5358751

