# Supplementary material for: Prefrontally mediated inhibition of memory systems in dissociative amnesia
Source: Psychol Med. 2025 Jan 8;54(16):4779–87. doi: 10.1017/S0033291724003040 (PMC11779556; doi:10.1017/S0033291724003040)

# **Supplementary material**

## 2.1. DCM results shown separately for each patient.

Model evidence for each of the 4 modulatory families:

**Patient 1 Patient 2 Both**


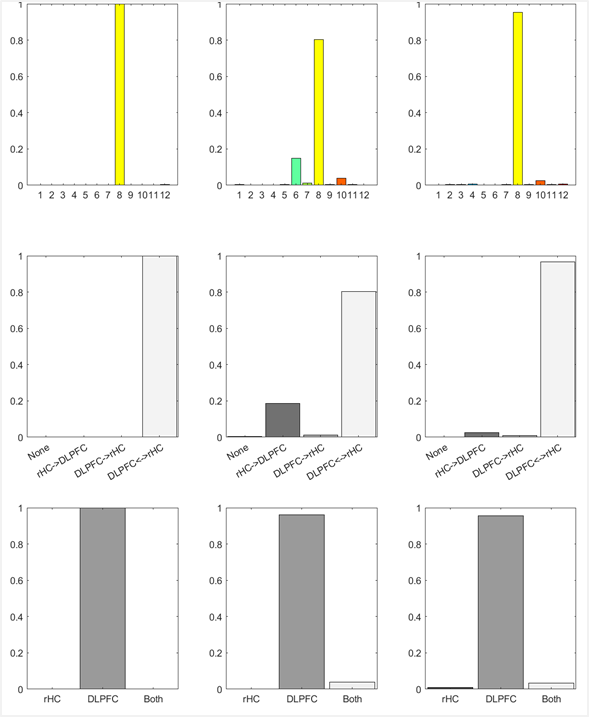

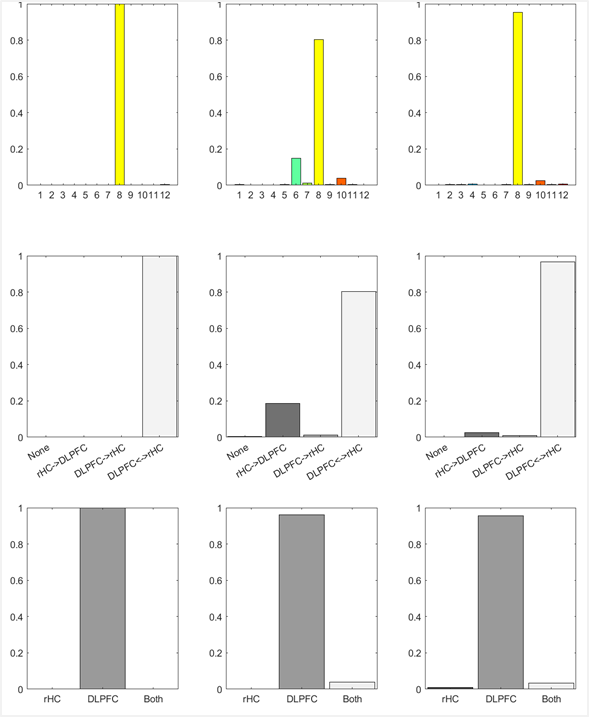

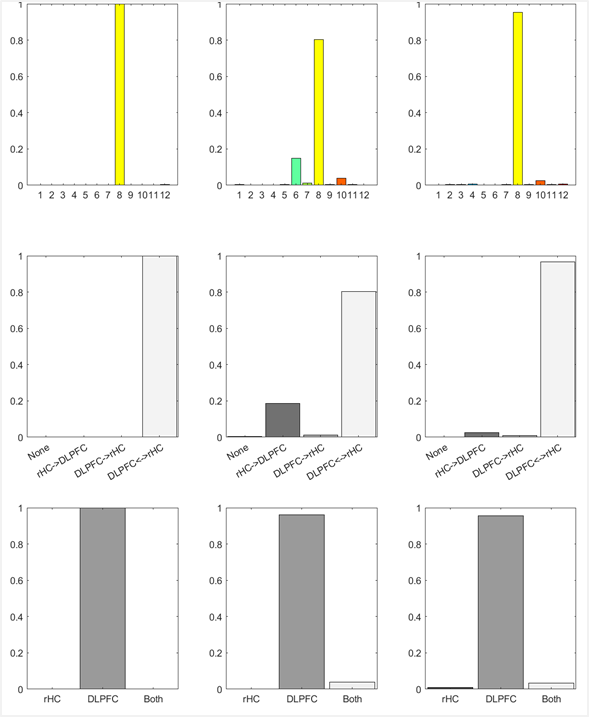


Model evidence for each of the 3 driving input families

**Patient 1 Patient 2 Both**


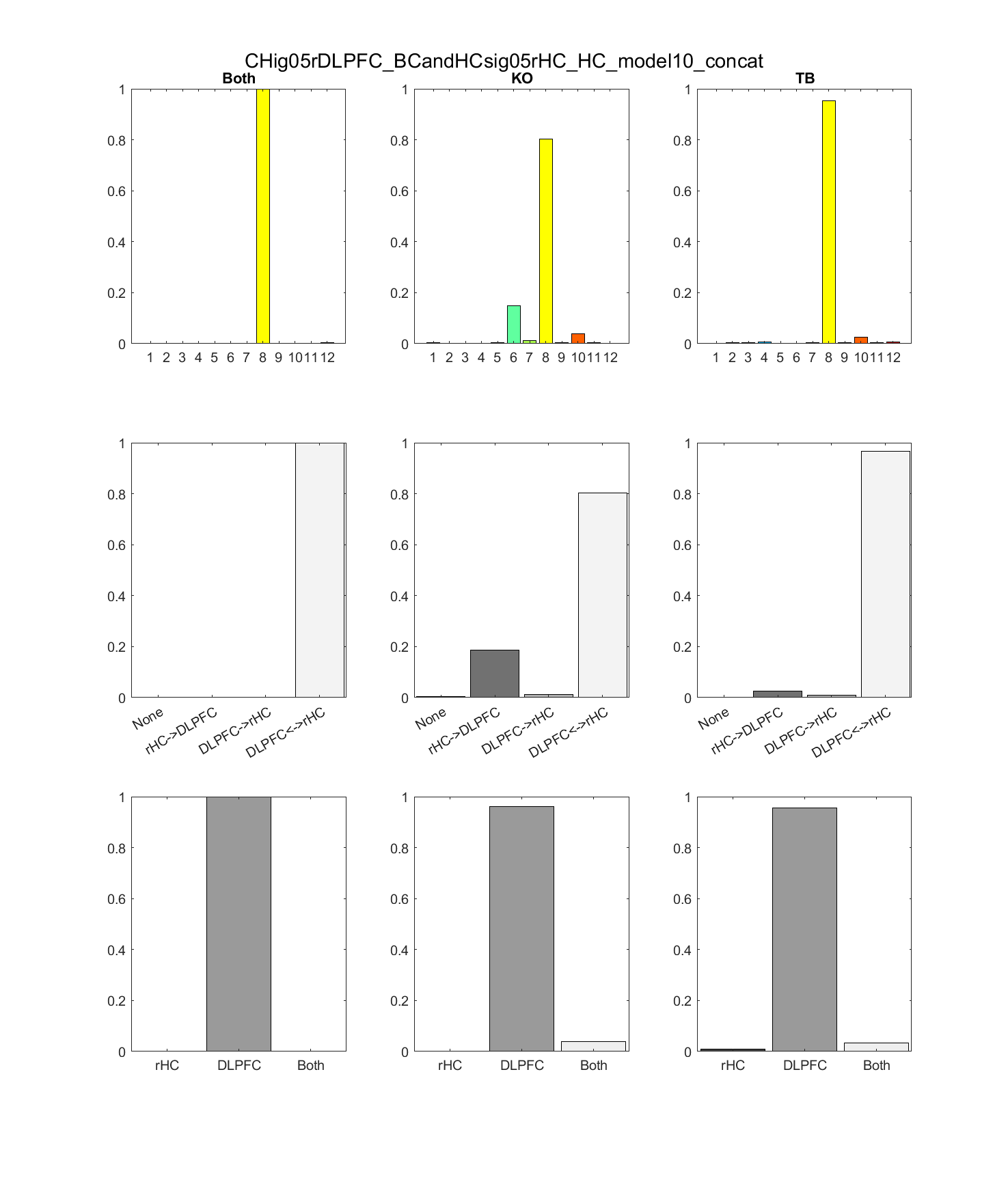

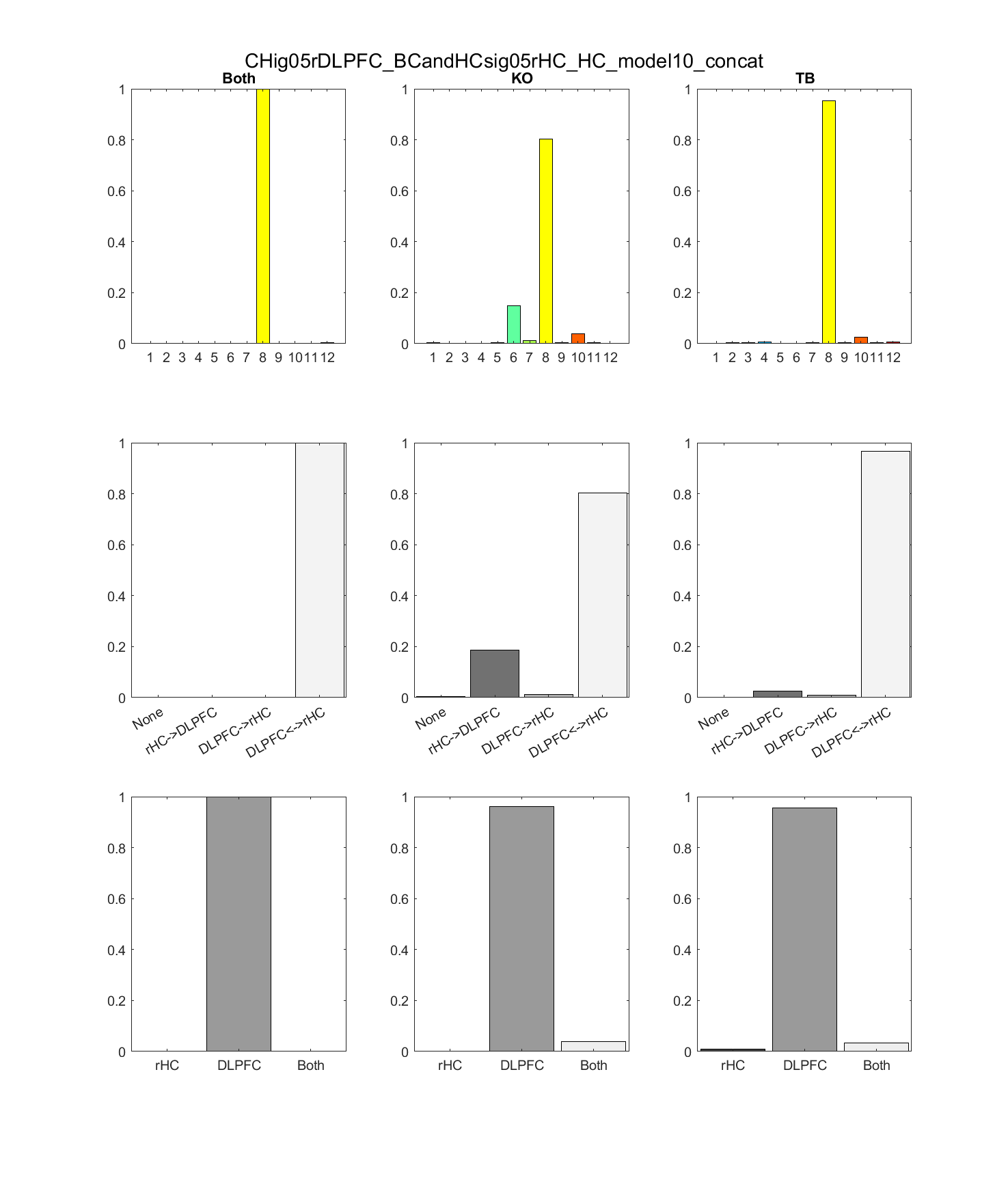

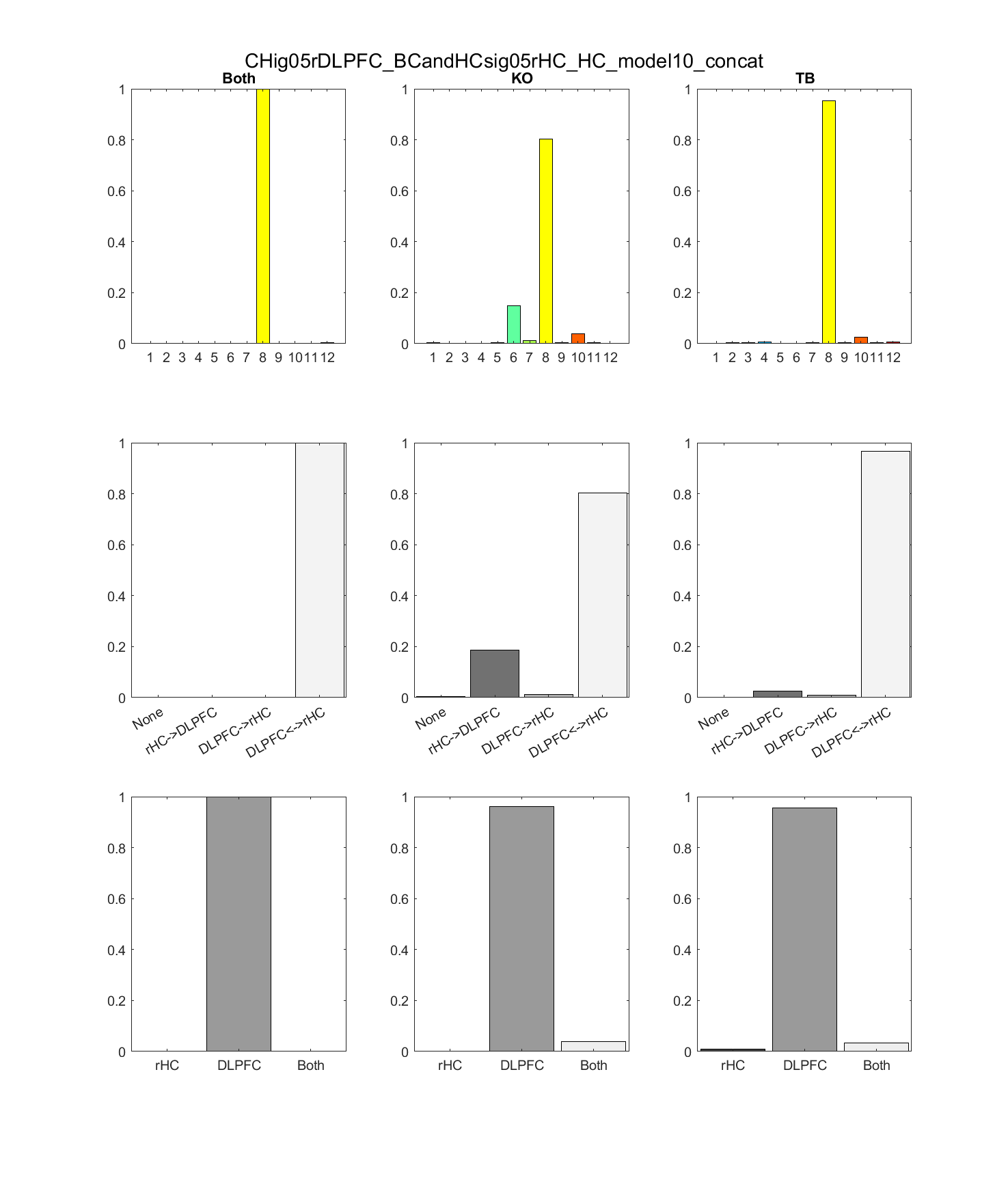


Model evidence across all 12 models

**Patient 1 Patient 2 Both**


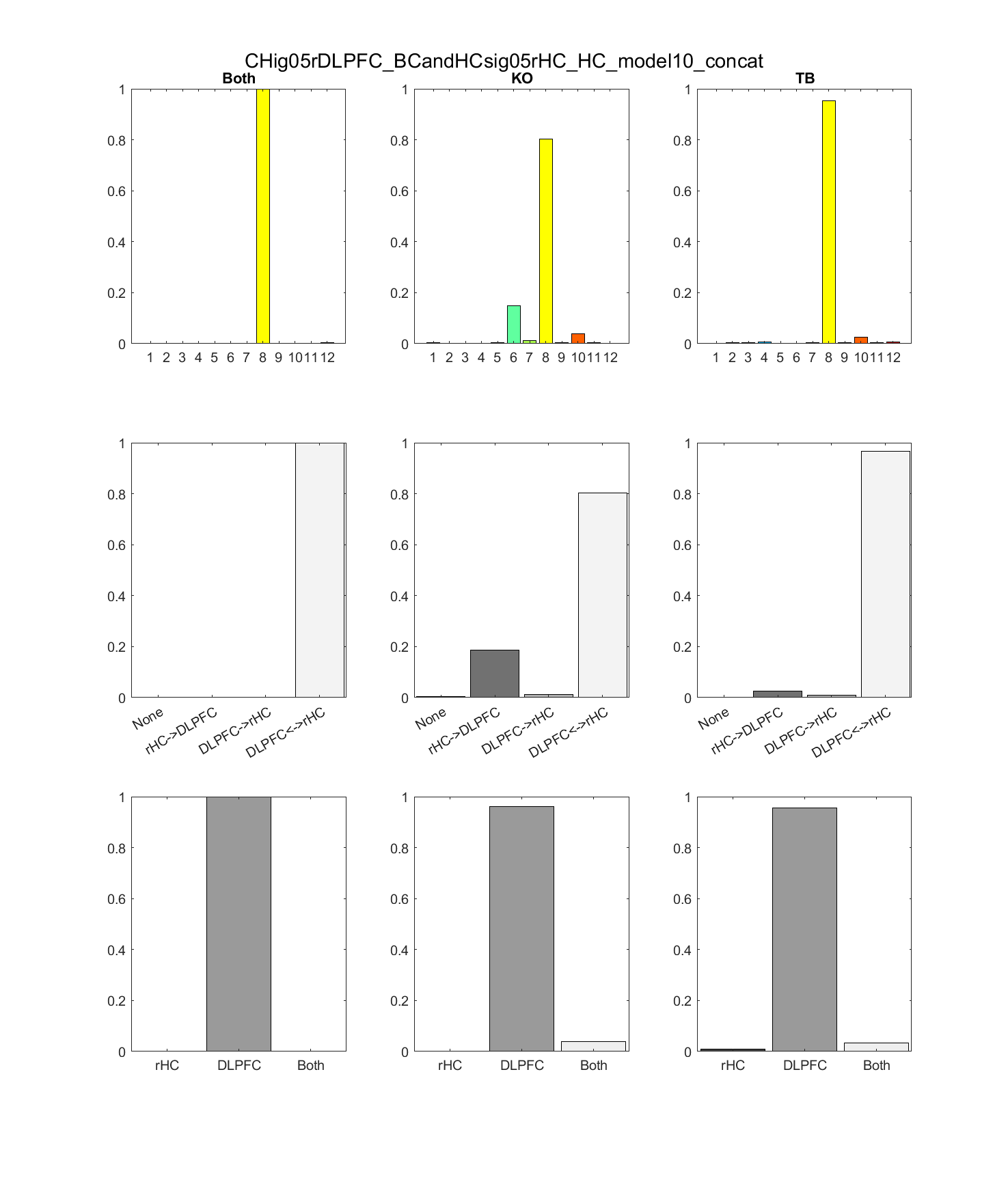


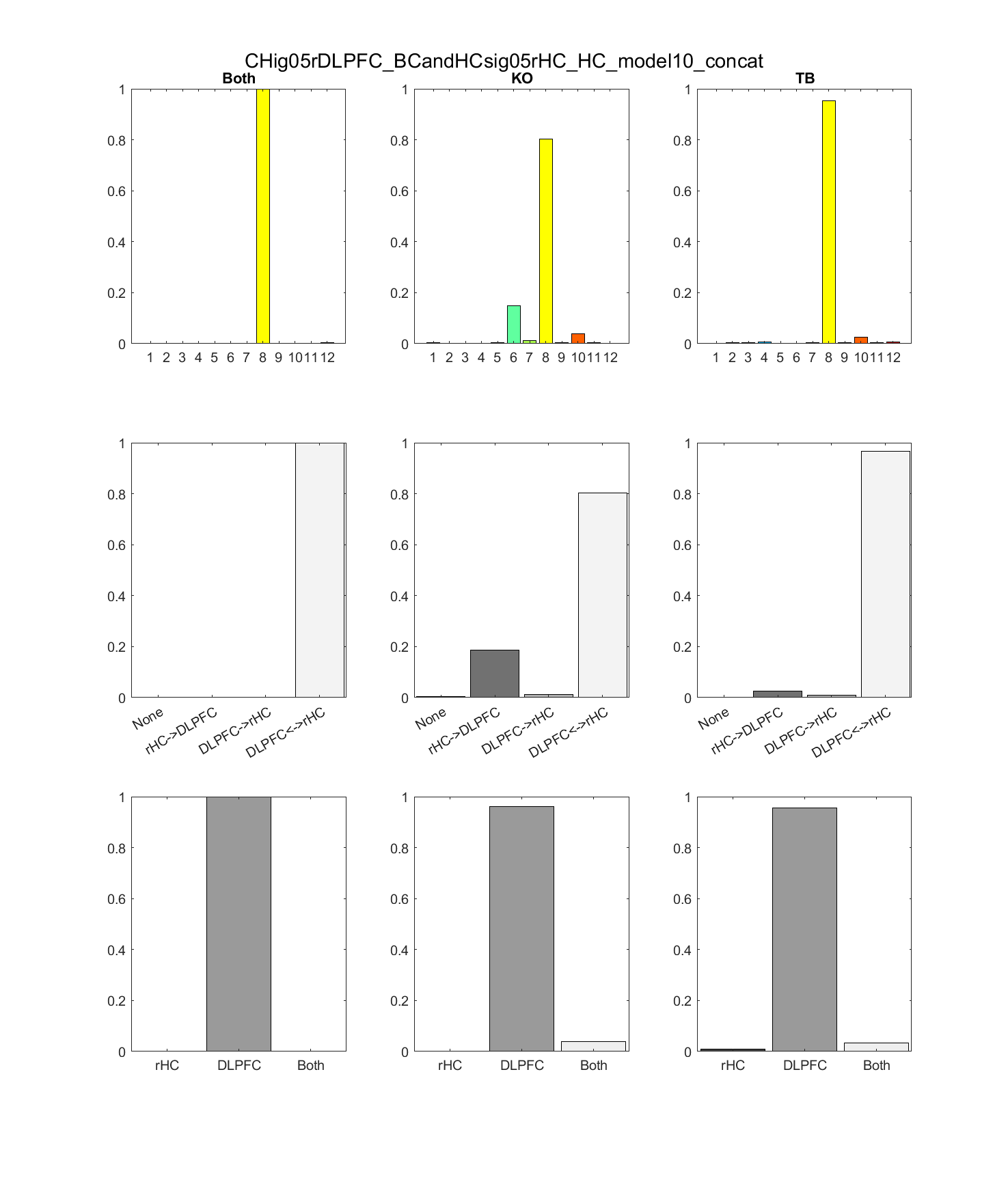

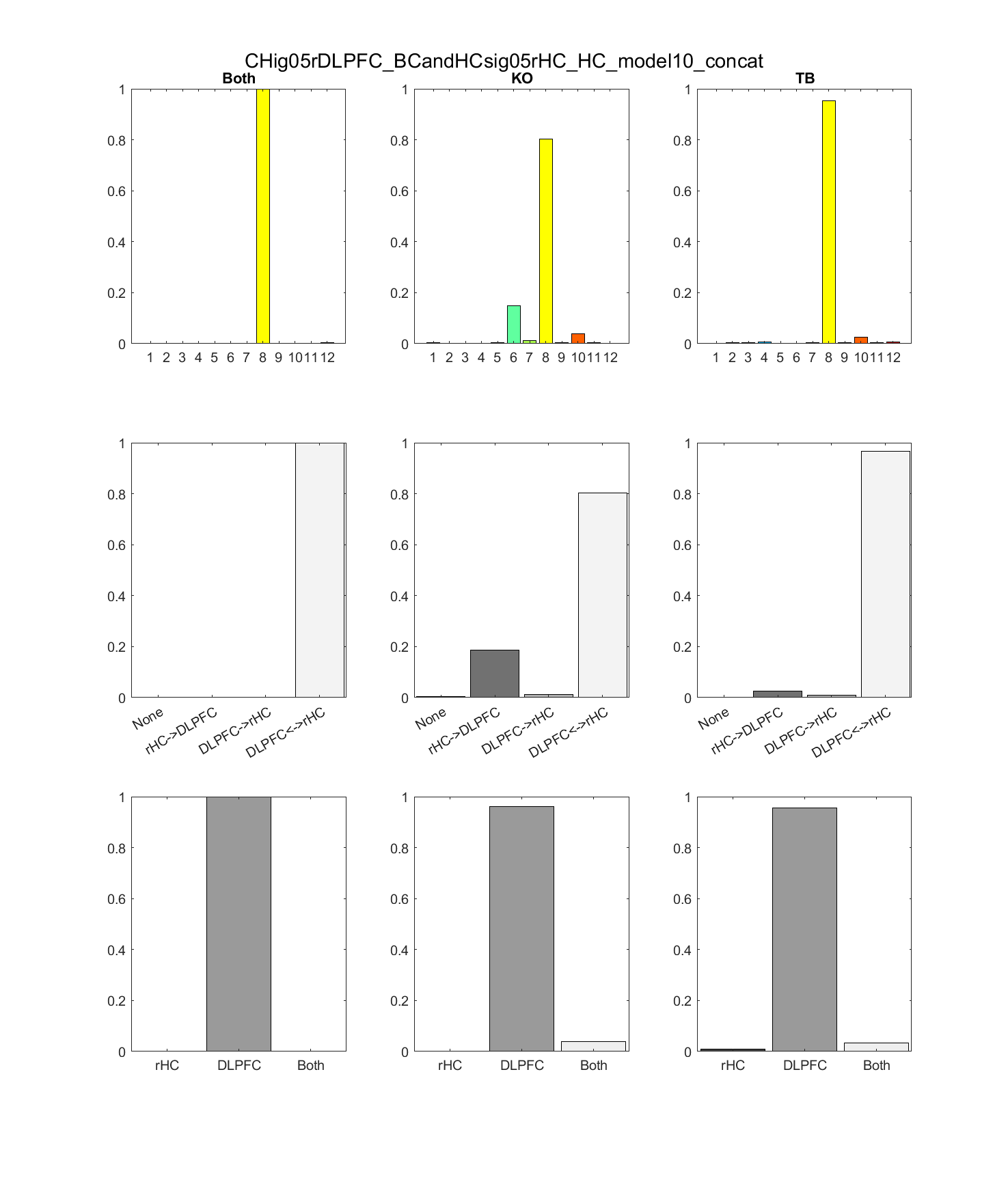


Coupling parameters

**Patient 1 Patient 2 Both**


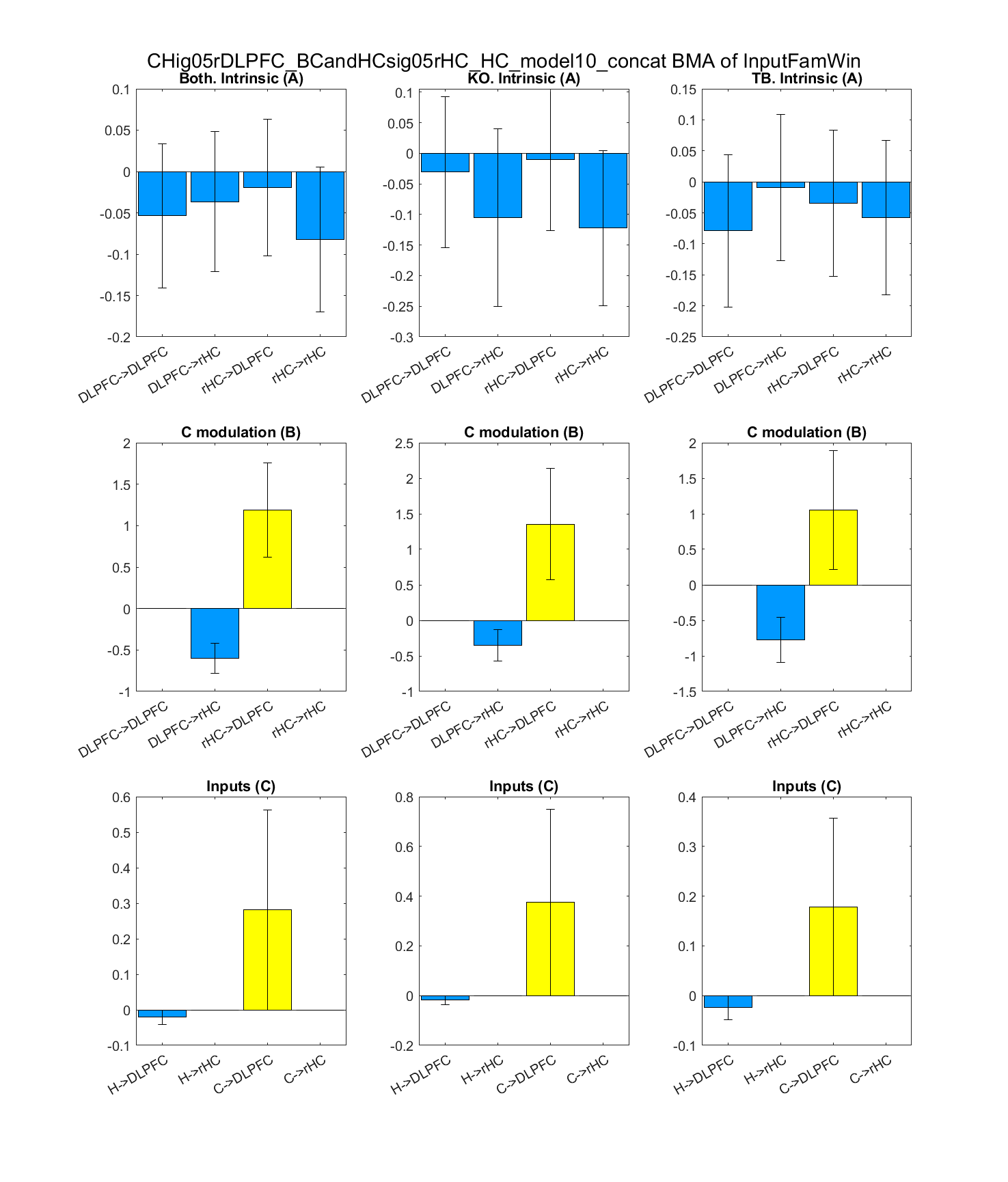

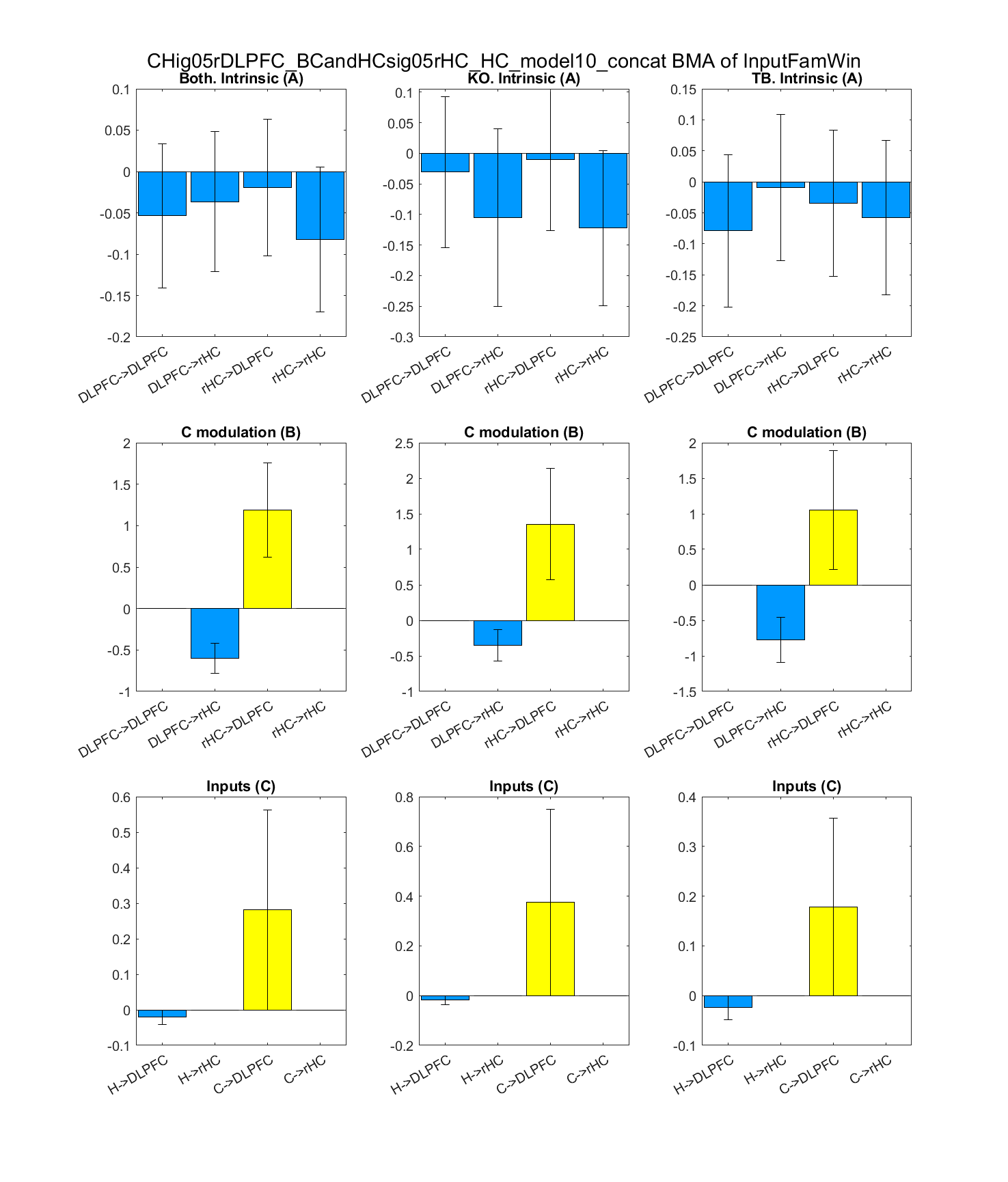

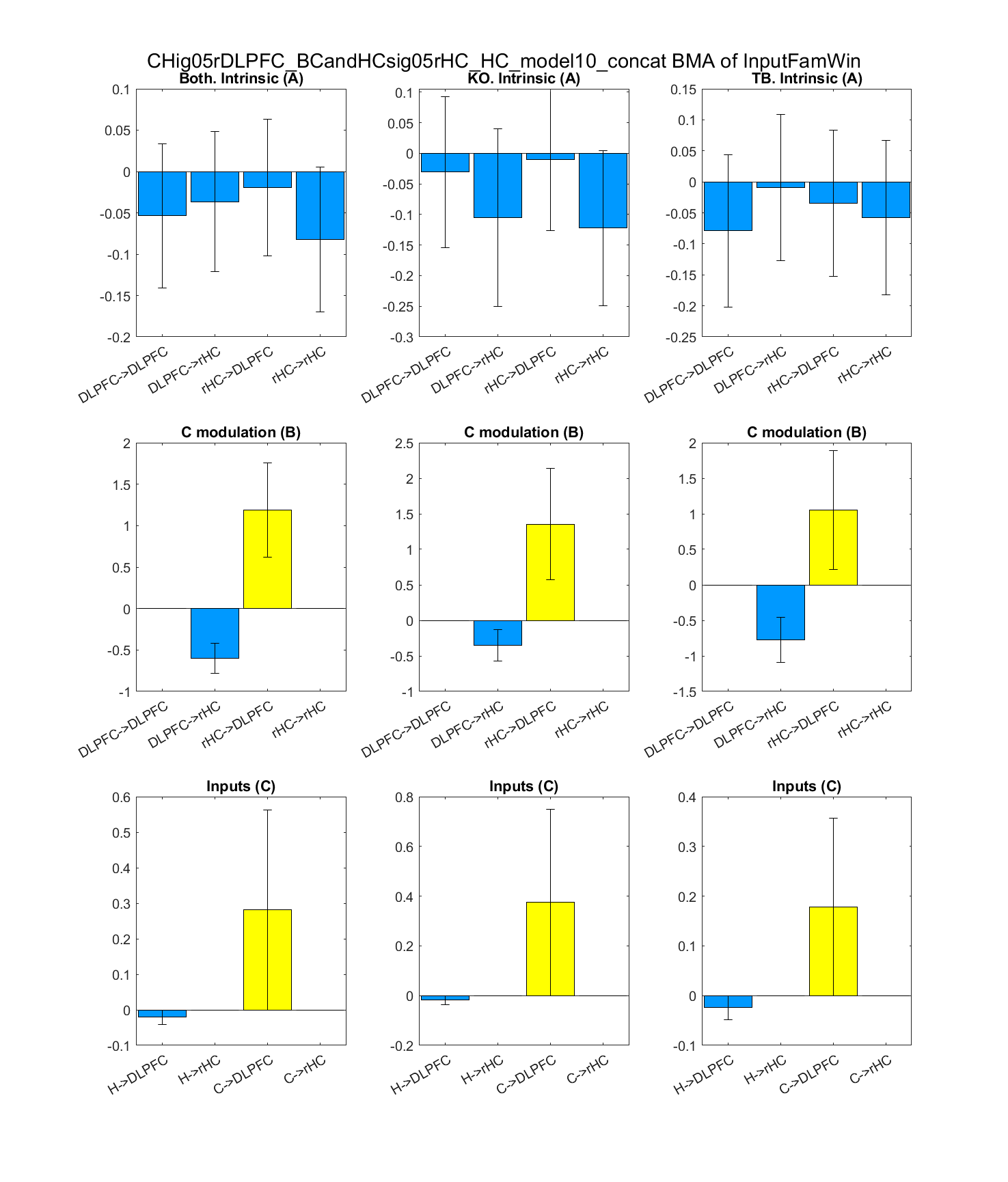

Supplement: Marsh et al. supplementary material [file S0033291724003040sup001.docx]
